# 1,2,3,4,6‐Penta‐O‐galloyl‐β‐d‐glucose modulates perivascular inflammation and prevents vascular dysfunction in angiotensin II‐induced hypertension

**DOI:** 10.1111/bph.14583

**Published:** 2019-03-14

**Authors:** Tomasz P. Mikolajczyk, Ryszard Nosalski, Dominik S. Skiba, Joanna Koziol, Magdalena Mazur, Amauri S. Justo‐Junior, Paulina Kowalczyk, Zofia Kusmierczyk, Agata Schramm‐Luc, Kevin Luc, Pasquale Maffia, Delyth Graham, Anna K. Kiss, Marek Naruszewicz, Tomasz J. Guzik

**Affiliations:** ^1^ Institute of Infection, Immunity and Inflammation University of Glasgow Glasgow UK; ^2^ Department of Internal and Agricultural Medicine Jagiellonian University Medical College Krakow Poland; ^3^ Institute of Cardiovascular and Medical Sciences University of Glasgow Glasgow UK; ^4^ Department of Pharmacognosy and Molecular Basis of Phytotherapy Medical University of Warsaw Warsaw Poland; ^5^ Department of Pharmacy University of Naples Federico II Naples Italy

## Abstract

**Background and Purpose:**

Hypertension is a multifactorial disease, manifested by vascular dysfunction, increased superoxide production, and perivascular inflammation. In this study, we have hypothesized that 1,2,3,4,6‐penta‐*O*‐galloyl‐β‐d‐glucose (PGG) would inhibit vascular inflammation and protect from vascular dysfunction in an experimental model of hypertension.

**Experimental Approach:**

PGG was administered to mice every 2 days at a dose of 10 mg·kg^−1^ i.p during 14 days of Ang II infusion. It was used at a final concentration of 20 μM for in vitro studies in cultured cells.

**Key Results:**

Ang II administration increased leukocyte and T‐cell content in perivascular adipose tissue (pVAT), and administration of PGG significantly decreased total leukocyte and T‐cell infiltration in pVAT. This effect was observed in relation to all T‐cell subsets. PGG also decreased the content of T‐cells bearing CD25, CCR5, and CD44 receptors and the expression of both monocyte chemoattractant protein 1 (CCL2) in aorta and RANTES (CCL5) in pVAT. PGG administration decreased the content of TNF^+^ and IFN‐γ^+^ CD8 T‐cells and IL‐17A^+^ CD4^+^ and CD3^+^CD4^−^CD8^−^ cells. Importantly, these effects of PGG were associated with improved vascular function and decreased ROS production in the aortas of Ang II‐infused animals independently of the BP increase. Mechanistically, PGG (20 μM) directly inhibited CD25 and CCR5 expression in cultured T‐cells. It also decreased the content of IFN‐γ^+^ CD8^+^ and CD3^+^CD4^−^CD8^−^ cells and IL‐17A^+^ CD3^+^CD4^−^CD8^−^ cells.

**Conclusion and Implication:**

PGG may constitute an interesting immunomodulating strategy in the regulation of vascular dysfunction and hypertension.

**Linked Articles:**

This article is part of a themed section on Immune Targets in Hypertension. To view the other articles in this section visit http://onlinelibrary.wiley.com/doi/10.1111/bph.v176.12/issuetoc

**Abbreviations**
CCR5: C–C chemokine receptor 5
CD: cluster of differentiation
MCP‐1: monocyte chemoattractant protein 1
MIP‐1: macrophage inflammatory protein 1
PGG: 1,2,3,4,6‐penta‐*O*‐galloyl‐β‐d‐glucose
pVAT: perivascular adipose tissue
RANTES: regulated on activation, normal T‐cell expressed and secreted
SNP: sodium nitroprusside
VAT: visceral adipose tissue, epididymal fat

**What is already known**

- PGG has a wide range of biological activities including anti‐cancer, anti‐inflammatory, anti‐oxidative effects.
- Little is known about the effect of PGG on hypertension or vascular inflammation.

**What this study adds**

- PGG is a candidate for targeting vascular inflammation and protecting from both oxidative stress and endothelial dysfunction
- PGG has direct effects on T cell activation and their perivascular recruitment;

**What is the clinical significance**

- Penta‐O‐galloyl‐β‐D‐glucose may provide opportunities for the effective treatment of vascular inflammation in cardiovascular disease and hypertension

## 1 INTRODUCTION

Vascular inflammation plays a pivotal role in the development and progression of hypertension and atherosclerosis (Guzik et al., 2007; Mikolajczyk et al., 2016; Skiba et al., 2017). Recent studies have shown the involvement of different leukocyte populations such as T‐cells (Guzik et al., 2007; Mikolajczyk et al., 2016), monocytes (Fujisawa et al., 2017; Wenzel et al., 2011), macrophages (Chan et al., 2012; Moore et al., 2015), dendritic cells (Kirabo et al., 2014), and NK cells (Kossmann et al., 2013) in hypertension (Guzik, Skiba, Touyz, & Harrison, 2017). **Angiotensin II** (Ang II) and other pro‐hypertensive stimuli induce immune cell activation and infiltration to the adventitia and peri‐adventitial fat (Guzik et al., 2007). These cells can produce a number of pro‐inflammatory cytokines and chemokines accelerating inflammation and contributing to vascular disease in hypertension (Guzik et al., 2007; Kusters, Lutgens, & Seijkens, 2018; Mikolajczyk et al., 2016). While a myriad of antihypertensive agents is available (Nguyen, Dominguez, Nguyen, & Gullapalli, 2010), only a few partially target these inflammatory mechanisms (Hermann & Ruschitzka, 2006). Current antihypertensive drugs, including those which target the Ang II system, are effective in controlling BP only in a subset of patients; however, they do not provide sufficient prevention of target organ damage in these individuals (Wright et al., 2002). Therefore, a more comprehensive way of targeting the pathology of hypertension is required, rather than solely focusing on BP regulation (Williams et al., 2018). This indicates a need for targeting the pathomechanisms involved in target organ damage including endothelial dysfunction, oxidative stress, and inflammation. The combination of a current antihypertensive agent with an anti‐inflammatory compound, which targets the immune system, would be more efficacious in preventing tissue damage. Suppression of vascular inflammation processes may inhibit or delay the progression of both hypertension and atherosclerosis. While systemic immunomodulation using anti‐cytokine treatment has recently been provided with an important proof‐of‐concept, it is imperative to identify novel agents that lack strong systemic immunosuppressive properties (Ridker et al., 2017). Many natural compounds derived from plants have shown anti‐inflammatory activity. The naturally occurring 1,2,3,4,6‐penta‐*O*‐galloyl‐β‐d‐glucose (PGG) is a polyphenolic compound highly enriched in *Oenothera paradoxa* (Jaszewska, Kosmider, Kiss, & Naruszewicz, 2009; Kiss, Derwinska, Dawidowska, & Naruszewicz, 2008; Kiss, Filipek, Czerwinska, & Naruszewicz, 2010). PGG has a wide range of biological activities including anticancer, anti‐diabetic, anti‐inflammatory, anti‐oxidative, anti‐allergy, antiviral, and antibacterial effects (Cryan et al., 2013; Zhang, Li, Kim, Hagerman, & Lu, 2009). Little is known about the effect of PGG on hypertension or vascular inflammation. However, PGG was shown to reverse cholesterol transport by influencing the expression of scavenger receptors, suggesting that this polyphenolic component may represent a novel candidate in the prevention and treatment of atherosclerosis in humans (Zhao, Haller, & Ritsch, 2015). Therefore, we aimed to investigate the effect of i.p. PGG administration on perivascular inflammation, vascular dysfunction, and BP elevation in an experimental model of Ang II‐dependent hypertension.

## 2 METHODS

### 2.1 Isolation of PGG

PGG was isolated from defatted seed extract of *O. paradoxa* Hudziok obtained from Agropharm S. A. (Tuszyn, Poland). One hundred grams of the seeds were dissolved in water (500 ml) and extracted with ethyl acetate (3 × 500 ml). Ethyl acetate fractions were dried under vacuum at 45 °C (giving a residue of ~10 g). The ethyl acetate extract was further fractionated on a polyamide column (5 cm × 8 cm; particle size 0.05–0.16 mm, Carl Roth, Germany) and eluted with acetone–water (7:3 [vol:vol]). One hundred fractions (50 ml each) were collected and pooled into 1A–6A main fractions based on their polyphenolic profile. PGG presence was monitored using HPLC. Fraction 5A (675 mg) was further subfractionated on a Toyopearl HW‐40, fine grade column (2.5 cm × 35 cm; Tosoh, Japan) using methanol–water (7:3 [vol:vol]) and acetone–water (7:3 [vol:vol]) as eluents. One hundred twenty fractions (10 ml each) were collected. Fractions 111–119 were separated again, eluted with acetone–water (4:6 [vol:vol]) as eluent to obtain a compound of purity >95% (390 mg). Chromatograms of crude seed extract and isolated PGG are presented in Figure S1.

### 2.2 Characterization of PGG

Off‐white powder; UV, λ max 281 nm; ESI‐MS (negative ion mode) *m*/*z* 939.0 [M‐H]^−^. ^1^H NMR (MD3OD) glucose moiety: δ 6.46 (1H, d, J = 8 Hz, H‐1), 6.20 (1H, t, H‐3), 5.85 (1H, m, H‐4), 4.78 (1H, m, H‐5), 4.51 (1H, m, H‐6). Galloyl moieties: δ 7.16, 7.18, 7.23, 7.25, 7.32 (each 2H, s). ^13^C NMR (MD3OD) glucose moiety: 93.85 (C‐1), 74.45 (C‐5), 74.13 (C‐3), 72.21 (C‐4), 69.82 (C‐2), 63.15 (C‐6). Galloyl moieties: 167.99, 167.35, 167.08, 166.98, 166.28 (carbonyl group signals), 146.63, 146.53, 146.50, 146.44, 146.34 (C‐3, C‐5), 141.00, 140.53, 140.48, 140.27, 140.14 (C‐4), 121.00, 120.31, 120.17, 120.13, 119.62 (C‐1), 110.63, 110.48, 110.42, 110.40, 110.35 (C‐2, C‐6). ^1^H and ^13^C spectra of PGG are presented in Figure S2.

### 2.3 Animals

Male C57BL/6J (RRID:IMSR_JAX:000664, Bar Harbor, ME, USA) mice (*n* = 70) were obtained from Jackson Laboratory. Mice were housed in controlled 12‐hr light/dark conditions at a constant temperature (21 ± 3 °C) with ad libitum access to water and standard diet (rat and mouse no. 1 maintenance diet, Special Diet Services). Twelve‐week‐old mice underwent either sham (buffer) or Ang II (490 ng·min^−1^·kg^−1^ s.c.) treatment for 14 days, using an osmotic minipump (Alzet Model 2002, Alzet Corporation, CA) as previously described (Mikolajczyk et al., 2016). This model of experimental hypertension in C57BL/6 mice has been well characterized previously (Guzik et al., 2007). Surgical procedures were performed under general anaesthesia by using isoflurane (3–5% for induction and 1.5–3% for maintenance of anaesthesia). PGG 10 mg·kg^−1^ was injected i.p. every 2 days, as previously described (Huh et al., 2005), starting 1 week before minipump implantation. PGG was initially dissolved in 96% ethanol, and then, 0.9% saline solution was added. The final concentration of ethanol was 7%. As a placebo, we used 0.9% saline solution with 7% ethanol. Mice were randomly assigned to either sham or Ang II and placebo or PGG‐treated groups. Individual mice were assigned numbers during randomization, therefore, data analysis for all subsequent endpoints was blinded for the treatment assignment groups. Placebo was administered to control animals. All animals underwent non‐invasive BP measurement by tail‐cuff plethysmography (Visitech bp 2000 bp Analysis System), following a 1‐week period of adaptation before surgery. Two weeks after minipump implantation, mice were killed, and aorta and adipose tissue (perivascular and visceral fat) were collected. All animal procedures were approved by Local Ethics Committee no. 1 in Krakow (Poland; permission no. 16/2012) and by the Home Office according to the Animals (Scientific Procedures) Act 1986 (project licence 60/9021). Animal studies are reported in compliance with the ARRIVE guidelines (Kilkenny et al., 2010) and with the recommendations made by the *British Journal of Pharmacology*.

### 2.4 Vascular reactivity measurements

Isometric tension studies in response to the endothelium‐dependent and endothelium‐independent vasodilators, **ACh** and **sodium nitroprusside (SNP)**, were performed following preconstriction with 1 μM PGF_**2α**_ in 3‐ to 4‐mm segments of aorta using tissue organ bath system 750TOBS (Danish Myo Technology) filled with Krebs solution (in mM: 124 NaCl, 4.6 KCl, 2.5 CaCl_2_, 1.2 MgSO_4_, 1.2 KH_2_PO_4_, 0.01 EDTA, 23 NaHCO_3_, and 11 glucose) as reported previously (Mikolajczyk et al., 2016; Siedlinski et al., 2017). All relaxation responses are expressed as a percentage of the PGF_2α_ preconstriction. The level of the preconstriction response to PGF_2α_ was matched between groups to the level of ~80% of maximal constriction. Moreover, PGG did not affect vascular contractility in response to PGF_2α_ in sham and Ang II‐infused animals.

### 2.5 Analysis of leukocytes in adipose tissue

For analysis of immune cells in adipose tissue compartments, perivascular adipose tissue (pVAT) or epididymal (visceral adipose tissue [VAT]) fat was digested using collagenase type XI (125 U·ml^−1^), collagenase type IS (450 U·ml^−1^), and hyaluronidase IV‐S (60 U·ml^−1^), which had been dissolved in PBS containing calcium and magnesium for 20 min at 37 °C, with regular agitation. Afterwards, digestion was stopped by addition of ice‐cold PBS with 20% FBS. The digested tissue was then passed through a 70‐μm sterile cell strainer (Falcon; BD Biosciences, San Jose, CA) to yield a single‐cell suspension. Cells were washed twice in PBS with 1% FBS, resuspended and counted; 0.5 × 10^6^ cells were stained with fluorophore‐ conjugated monoclonal antibodies: anti‐CD45‐FITC (Clone 30‐F11, BD Biosciences, San Jose, CA, USA), anti‐CD3e‐APC (Clone 145‐2C11, BD Biosciences), anti‐CD4‐APHC7 (Clone GK1.5, BD Biosciences), anti‐CD8a‐PERCP (Clone 53–6.7, BD Biosciences), anti‐CD25‐PE (Clone PC61.5, eBioscience, San Diego, CA, USA), anti‐CD195‐PE (Clone HM–**CCR5** [7A4], eBioscience), anti‐CD44‐FITC (Clone IM7, BD Biosciences), anti‐F4/80‐APC (Clone BM8, eBioscience), and anti‐CD11b‐PE (Clone M1/70, BD Biosciences). After 20 min of staining on ice, cells were washed twice in PBS with 1% FBS and were analysed using a BD FACSVerse™ flow cytometer with BD FACSuite™ software (BD Biosciences). T‐cells were analysed from CD45 positive cells based on CD3 expression and Side scatter (SSC) signal. Next, within the CD3 gate, CD4, CD8, and CD3^+^CD4^–^CD8^–^ cells were analysed. Macrophages were defined as the F4/80^+^CD11b^+^ population. For each experiment, we performed fluorescence minus one controls for each fluorophore to establish gates. Dead cells were eliminated from the analysis using BD Horizon™ Fixable Viability Stain 510 (BD Biosciences). Data were analysed by FlowJo (RRID:SCR_008520) software (FlowJo, Ashland, OR).

### 2.6 Assessment of intracellular cytokines

Splenocytes (1 × 10^6^ ) were suspended in RPMI 1640 medium (Gibco, Life Technologies, USA) with 10% FBS, 200‐mM l‐glutamine, and 5 mg ml^−1^ gentamicin (Sigma‐Aldrich, St. Louis, MO, USA) and were cultured without or with PGG in a final concentration of 20 μM for 30 min.

In parallel in vivo experiments, leukocytes isolated from pVAT from sham‐ and Ang II‐infused animals treated with PGG or placebo were studied. Next, cells were stimulated with leukocyte activation cocktail with BD Golgi Plug from BD Biosciences for 4 hr at 37 °C in a 5% CO_2_ humidified atmosphere. Cells were then washed in PBS with 1% FBS and stained with monoclonal antibodies: anti‐CD3e‐APC (Clone 145‐2C11, BD Biosciences), anti‐CD4‐APHC7 (Clone GK1.5, BD Biosciences), and anti‐CD8a‐PERCP (Clone 53–6.7, BD Biosciences). Following washing, fixation and permeabilization solution (eBioscience) was used for 30 min on ice. After fixation/permeabilization, cells were washed with permeabilization buffer (eBioscience) and stained for 20 min with monoclonal antibodies: anti‐TNF‐PE (Clone MP6‐XT22, BD Biosciences), anti‐IL‐17A‐PE (Clone eBio17B7, eBioscience), anti‐IL‐17A‐PE‐Cy7 (Clone eBio17B7, eBioscience), and anti‐IFN‐γ‐FITC (Clone XMG1.2, BD Biosciences). Dead cells were eliminated from the analysis using BD Horizon™ Fixable Viability Stain 510 (BD Biosciences). After additional washes, cells were analysed using a BD FACSVerse™ flow cytometer.

### 2.7 Assessment of T‐cell activation markers in vitro

To evaluate the effect of PGG on the expression of activation markers on the surface of T‐cells, anti‐CD3 plates were used; 96‐well plates were coated with anti‐CD3 monoclonal antibodies (Clone 145‐2C11; Thermo Fisher Scientific, Waltham, MA, USA, Cat# 16‐0031‐81, RRID:AB_468846). Splenocytes were isolated from C57Bl/6J mice and then were suspended in RPMI 1640 medium (Gibco, Life Technologies, Grand Island, NY, USA) with 10% FBS, 200‐mM l‐glutamine, and 5 mg ml^−1^ gentamicin (Sigma‐Aldrich) and were cultured with or without PGG (20 μM) on anti‐CD3‐coated plates for 20 hr. After being washed in PBS with 1% FBS, they were stained with monoclonal antibodies to identify T‐cell subsets (see above) and with activation marker monoclonal antibodies: anti‐CD25‐PECY7 (Clone PC61, BD Biosciences), anti‐CD195‐PE (Clone HM‐CCR5 [7A4], eBioscience), and anti‐CD44‐FITC (Clone IM7, BD Biosciences). Dead cells were eliminated from the analysis using BD Horizon™ Fixable Viability Stain 510 (BD Biosciences). After being stained the cells were washed in PBS with 1% FBS and were collected using a BD FACSVerse™ flow cytometer.

### 2.8 Detection of vascular superoxide production

Aortic segments were equilibrated in oxygenated Krebs‐HEPES (in mM: NaCl 99.0, NaHCO_3_ 25, KCl 4.7, KH_2_PO_4_ 1.0, MgSO_4_ 1.2, glucose 11.0, CaCl 22.5, and Na‐HEPES 20.0) buffer for 15 min at 37 °C. Lucigenin‐enhanced chemiluminescence (5 μmol·L^−1^) from intact vessels was measured in buffer (2 ml) containing lucigenin at 37 °C using FB12 Tube Luminometer (Tritertek Berthold) as previously described (Sagan et al., 2012; Siedlinski et al., 2017). Superoxide production was expressed as RLU·s·mg^−1^ of dry weight as described before (Siedlinski et al., 2017).

### 2.9 Cell culture and gene expression measurements

SW 872 cells (ATCC, USA) were grown at 37 °C in a 5% CO_2_ atmosphere in DMEM supplemented with 10% FBS, 50 U·mL^−1^ penicillin, and 50 U·mL^−1^ streptomycin. For SW872 differentiation to adipocytes, medium was replaced with 10% FBS DMEM with **insulin** (1 μM; Cell Applications, San Diego, CA), **dexamethasone** (0.25 μM; Sigma‐Aldrich), and **IBMX** (0.5 mM; Sigma‐Aldrich) for 48 hr. Medium was replaced with 10% FBS DMEM with insulin (1 μM) for another 48 hr. For the next 4 days, medium was replaced with 10% FBS DMEM. A day before stimulation, cells were starved in 1% FBS DMEM and then were stimulated with 50 μM PGG for 24 hr. Total RNA was obtained from cells using RNeasy Mini Kit (Qiagen, Hilden, Germany) and was measured by Nanodrop 1000 (Thermo Fisher Scientific). Reverse transcription of 1 μg RNA was performed using High Capacity cDNA Reverse Transcription Kit (Applied Biosystems). mRNA expressions of chosen genes were analysed using Fast SYBR® Green Master Mix (Thermo Fisher Scientific) and designed primers (TNF, forward primer sequence [5′ → 3′] AGCCCATGTTGTAGCAAACC, reverse primer sequence [5′ → 3′] TGAGGTACAGGCCCTCTGAT; *MCP‐1* *(CCL2)* forward primer sequence [5′ → 3′] CCCCAGTCACCTGCTGTTAT, reverse primer sequence [5′ → 3′] AGATCTCCTTGGCCACAATG; *RANTES* *(CCL5)* forward primer sequence [5′ → 3′] CGCTGTCATCCTCATTGCTA, reverse primer sequence [5′ → 3′] GAGCACTTGCCACTGGTGTA (Eurofins, Luxembourg). Reactions were prepared and run on 384‐well plates on the QuantStudio™ 7 Flex Real‐Time PCR System with standard protocol. Calculations were made using QuantStudio™ Real‐Time PCR Software. Data were normalized to levels of GAPDH mRNA, and relative quantification was calculated in comparison to control.

### 2.10 Gene expression measurements in aortic and pVAT

Collected tissue was stored in RNAlater stabilization solution (Ambion, Thermo Fisher Scientific) until RNA isolation. Total RNA was obtained from cells using RNeasy Mini Kit (Qiagen) and was measured by Nanodrop 1000 (Thermo Fisher Scientific). Reverse transcription of 1 μg RNA was performed using High Capacity cDNA Reverse Transcription Kit (Applied Biosystems, Foster City, CA, USA). mRNA expressions of chosen genes were analysed using TaqMan assays for CCL2 (MCP‐1; Mm00441242_m1), CCL3 (Mm00441259_g1), CCL4 (Mm00443111_m1), and CCL5 (RANTES; Mm01302427_m1). Reactions were prepared and run on 384‐well plates on the QuantStudio™ 7 Flex Real‐Time PCR System with standard protocol. Calculations were made using QuantStudio™ Real‐Time PCR Software. Data were normalized to levels of Eukaryotic Translation Elongation Factor 2 (EEF2) (Mm05700170_g1) mRNA, and relative quantification was calculated in comparison to control.

### 2.11 Statistical analysis

For comparison of the effects of Ang II and PGG on parameters in different groups of mice, we used two‐way ANOVA with a Bonferroni post hoc test. For comparisons of vascular function in organ chamber experiments, repeated‐measures ANOVA was used.

For comparison of two groups, Student's paired *t* test was used. Values of *P* < 0.05 were considered significant. For statistical analysis, GraphPad PRISM version 6.0c was used. The data and statistical analysis comply with the recommendations of the *British Journal of Pharmacology* on experimental design and analysis in pharmacology.

### 2.12 Nomenclature of targets and ligands

Key protein targets and ligands in this article are hyperlinked to corresponding entries in http://www.guidetopharmacology.org, the common portal for data from the IUPHAR/BPS Guide to PHARMACOLOGY (Harding et al., 2018), and are permanently archived in the Concise Guide to PHARMACOLOGY 2017/18 (Alexander et al., 2017).

## 3 RESULTS

### 3.1 Effect of PGG on immune cell infiltration into pVAT during Ang II‐dependent hypertension

**Figure 1:**
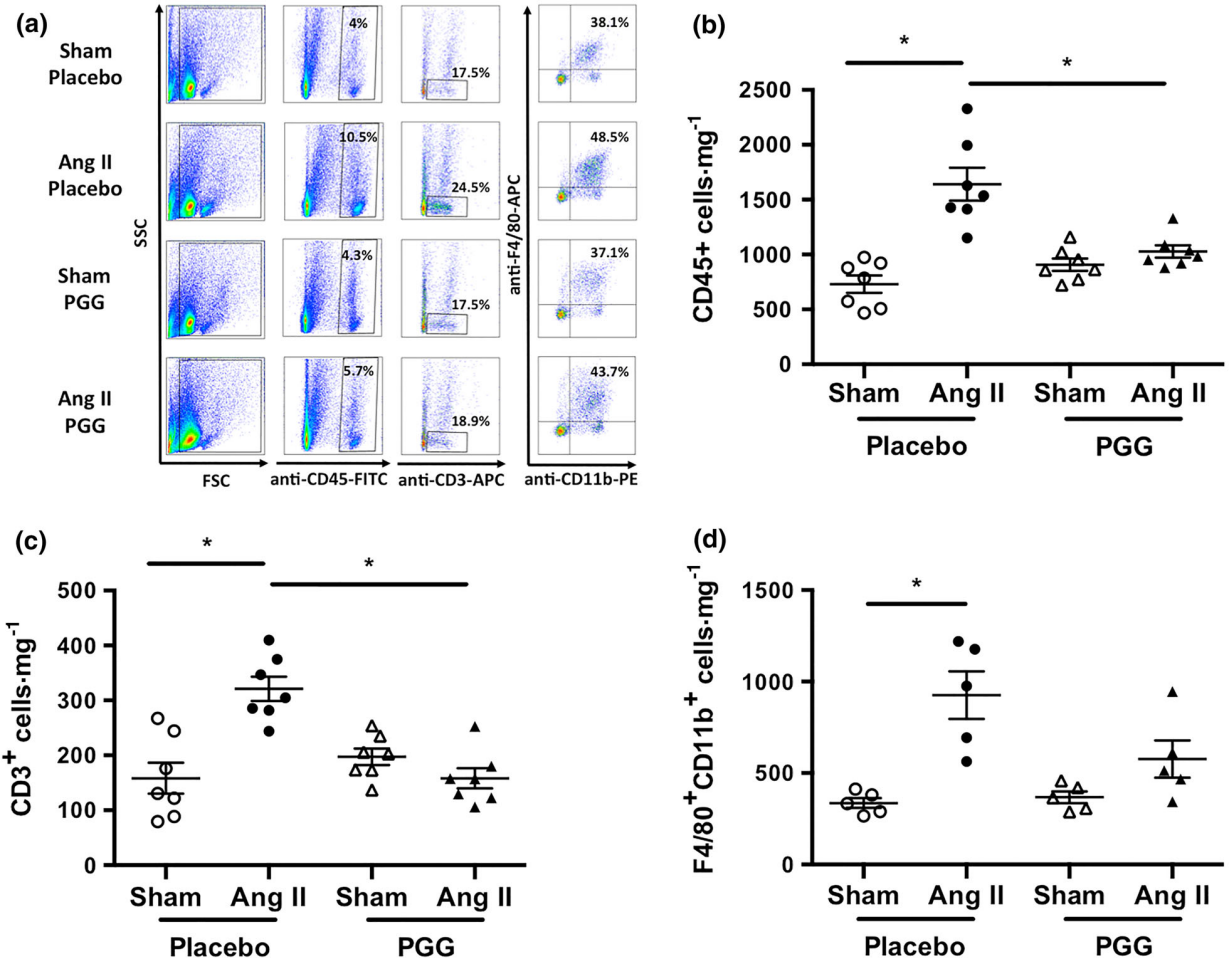
Effect of 1,2,3,4,6‐penta‐*O*‐galloyl‐β‐d‐glucose (PGG) on leukocyte infiltration in perivascular adipose tissue (pVAT) during Ang II‐dependent hypertension. Hypertension was induced by chronic 14‐day infusion of Ang II by osmotic minipump (490 ng·min^−1^·kg^−1^), and leukocytes were isolated from pVAT using enzymatic digestion. (a) Representative flow cytometric analysis of major leukocyte subpopulations in vascular stromal fraction isolated from pVAT of sham‐ and Ang II‐infused mice treated with PGG or placebo. (b) Effect of Ang II infusion and PGG administration on absolute numbers of CD45^+^ total leukocyte content in pVAT expressed per mg of tissue (*n* = 7). (c) and (d) Effect of Ang II infusion and PGG administration on CD3^+^ T‐cells (*n* = 7) and F4/80^+^CD11b^+^ macrophage (*n* = 5) content respectively. *P < 0.05

**Figure 2:**
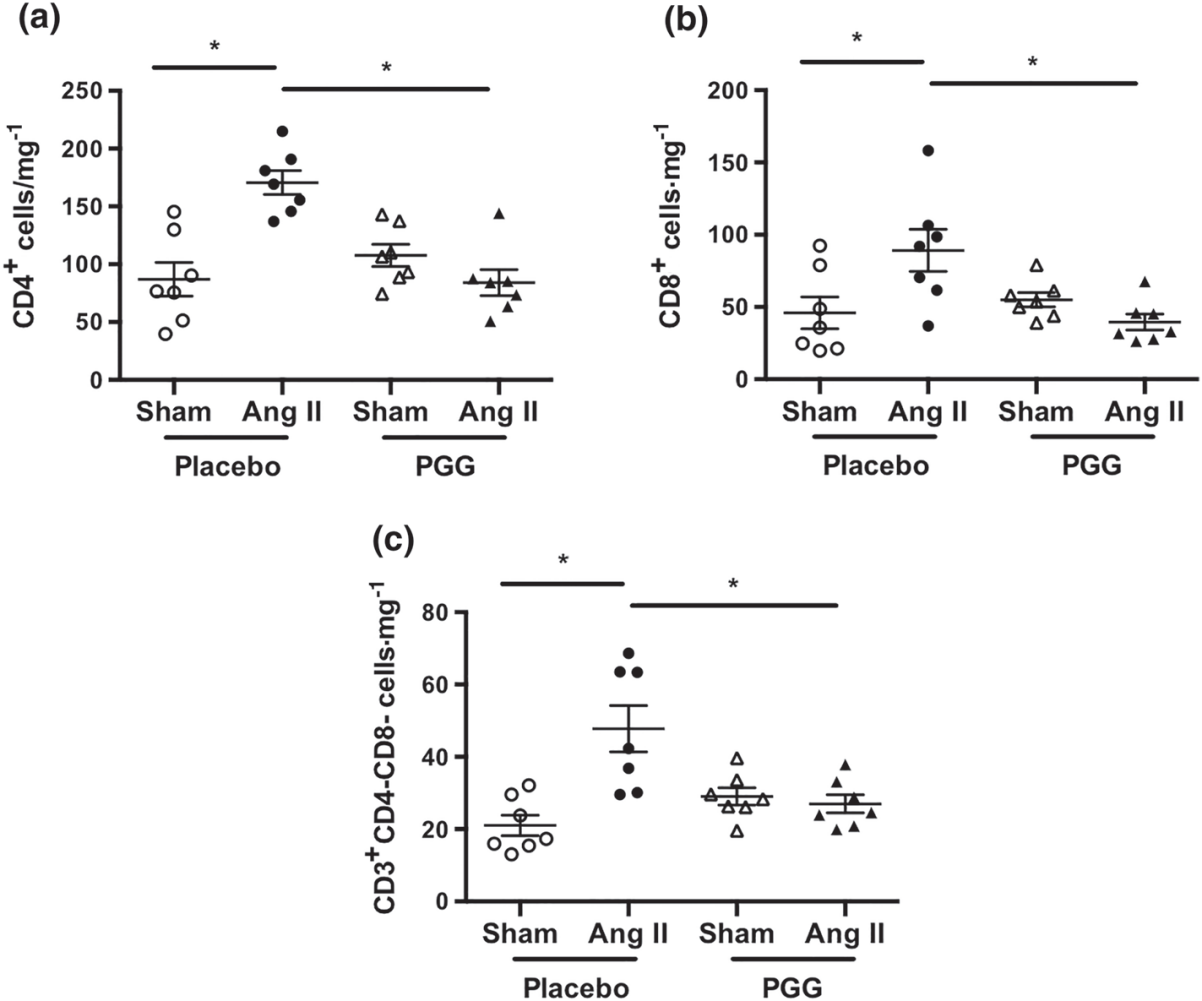
Effect of 1,2,3,4,6‐penta‐*O*‐galloyl‐β‐d‐glucose (PGG) on T‐cell subset infiltration in perivascular adipose tissue during Ang II‐dependent hypertension. Absolute number of lymphocytes (a) CD4^+^, (b) CD8^+^, and (c) CD3^+^CD4^−^CD8^−^ in perivascular adipose tissue expressed as cells·mg^−1^ of tissue (*n* = 7 each). *P < 0.05

The effect of PGG on leukocyte infiltration into the perivascular tissue was investigated in a mouse model of experimental hypertension. In line with previous findings, 14‐day administration of Ang II significantly increased the content of total leukocytes in pVAT (Figure 1a). Absolute number of leukocytes significantly increased following Ang II infusion (730 ± 79 vs. 1640 ± 150 cells·mg^−1^, *P* < 0.05; Figure 1b). Importantly, i.p. administration of PGG (every 2 days) 1 week prior to implantation of minipumps and during 2 weeks of Ang II infusion significantly reduced the total number of CD45^+^ cells in the pVAT in comparison to animals which did not receive this drug (1640 ± 150 vs. 1028 ± 57 cells·mg^−1^, *P* < 0.05). In summary, PGG prevented leukocyte infiltration of pVAT during Ang II infusion (Figure 1b). As identified before, CD3^+^ T‐cells were particularly increased following Ang II administration (158 ± 28 vs. 321 ± 22 cells·mg^−1^, *P* < 0.05) and PGG efficiently prevented this infiltration (321 ± 22 vs. 158 ± 18 cells·mg^−1^, *P* < 0.05; Figure 1a,c). It is important to note that we also observed a statistically significant change in F4/80 cell content in the placebo group and a lack of such an increase in PGG‐treated mice, indicating that an effect was also observed on recruitment of macrophages (Figure 1a,d). Further characterization has shown that all major T‐cell subsets, including CD4^+^, CD8^+^, and CD3^+^CD4^−^CD8^−^ cells (Figure 2a–c), were increased in Ang II‐treated mice, and PGG prevented these increases (Figure 2a–c).

The effect of Ang II infusion on the total number of leukocytes, T‐cells, and elevation of macrophages was particularly seen in pVAT but not in VAT (Figure S3a–c). Administration of PGG had no effect on the number of CD45^+^, CD3^+^, and F4/80^+^CD11b^+^ cells in VAT (Figure S3).

### 3.2 Effect of PGG on the content of activated T‐cells during Ang II‐dependent hypertension

**Figure 3:**
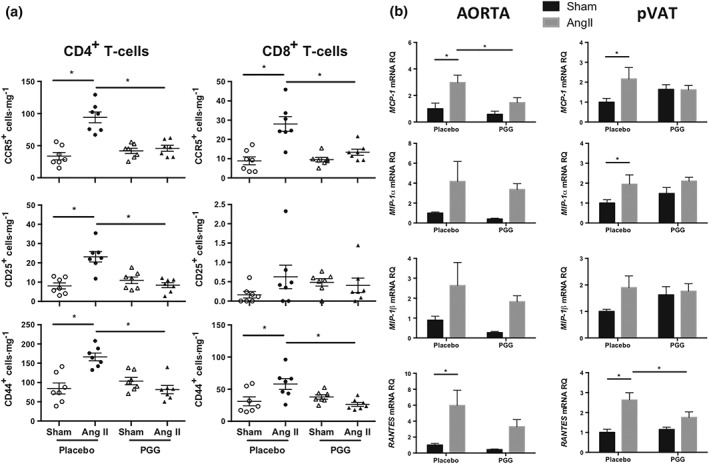
Effect of 1,2,3,4,6‐penta‐*O*‐galloyl‐β‐d‐glucose (PGG) on T‐cell infiltration in perivascular adipose tissue (pVAT) and the expression of selected genes in aorta and pVAT in Ang II‐dependent hypertension. (a) Absolute number of CD4^+^ and CD8^+^ T‐cells bearing CCR5, CD25, and CD44 in pVAT expressed as cells·mg^−1^ of tissue (*n* = 7 each). (b) Expression of MCP‐1 (CCL2), MIP‐1α (CCL3), MIP‐1β (CCL4), and RANTES (CCL5) in aorta and pVAT of sham and Ang II groups treated with PGG or placebo (*n* = 5 for sham and *n* = 6 for Ang II). *P < 0.05

As we had previously identified that the RANTES–CCR5 axis is essential for activated T‐cell recruitment in hypertension (Mikolajczyk et al., 2016), we next studied the effects of PGG on the expression of CCR5 and activation status of infiltrating T‐cells. Ang II‐dependent hypertension was associated with an increased number of CCR5 positive (both CD4^+^ and CD8^+^) T‐cells in pVAT (Figure 3a). Additionally, the number of CD25^+^CD4^+^, but not CD25^+^CD8^+^ T‐cells, was increased following Ang II infusion (Figure 3a). This effect was not seen in mice that had received PGG during Ang II administration (Figure 3a). Administration of PGG during Ang II‐dependent hypertension significantly decreased the number of CCR5^+^CD4^+^ and CCR5^+^CD8^+^ cells (Figure 3a). PGG also efficiently reduced the content of CD4^+^ cells bearing CD25 during the development of hypertension (Figure 3a). Ang II infusion increased the number of CD44^+^ (both CD4^+^ and CD8^+^) T‐cells, and PGG efficiently reduced the content of these cells when it was administered together with Ang II (Figure 3a).

### 3.3 PGG exhibits anti‐inflammatory effects in vitro

To investigate the effect of PGG on T‐cell activation in vitro, we cultured freshly isolated splenocytes on anti‐CD3‐coated plates in the absence or presence of PGG in the final concentration 20 μM. After 20 hr of cell culture, we observed that addition of PGG to the splenocytes slightly reduced the percentage of CCR5^+^CD3^+^ and CCR5^+^CD4^+^ cells. In addition, the mean intensity of fluorescence for CCR5 was decreased within CD4^+^ T‐cells (Table 1). PGG also decreased the mean intensity of fluorescence for CD25 in both CD4^+^ and CD8^+^ T‐cells but had no effect on the expression of hyaluronan receptor—CD44 (Table 1), confirming possible direct effects of PGG on T‐cell biology, which were observed in vivo.

**Table 1:** Effect of PGG (20 μM) on the expression of selected receptors on T‐cells

	CD3^+^		CD4^+^		CD8^+^		CD3^+^CD4^−^CD8^−^	
	Placebo	PGG	Placebo	PGG	Placebo	PGG	Placebo	PGG
CCR5 % (mean)	33 ± 1.3 (328 ± 17)	30.5 ± 0.6* (301 ± 4.4)	32 ± 1 (352 ± 22.5)	29 ± 1* (295 ± 4.3)*	32.9 ± 1,3 (311 ± 18)	30.7 ± 0.5 (295 ± 3.9)	36.7 ± 2 (305 ± 6.4)	35 ± 1.7 (308 ± 15)
CD25 % (mean)	46.4 ± 4 (2,144 ± 66)	45 ± 4 (1,785 ± 54)*	55.9 ± 5.6 (2,608 ± 147)	48.8 ± 4.4 (2,193 ± 69)*	40.7 ± 3.76 (1,770 ± 74)	41.5 ± 4 (1,420 ± 83)*	47.7 ± 3.8 (1,934 ± 100)	53 ± 3.8* (1,762 ± 57)
CD44 % (mean)	75 ± 2 (5,247 ± 276)	74 ± 1.9 (5,375 ± 270)	93 ± 1.16 (4,176 ± 321)	93 ± 1 (4,282 ± 238)	60 ± 2.8 (4,384 ± 159)	58 ± 2 (4,670 ± 289)	87.9 ± 2.4 (13,499 ± 813)	90 ± 1.7 (13,551 ± 814)

*Note*: Freshly isolated splenocytes were cultured on anti‐CD3‐coated plates in the presence of PGG (final concentration 20 μM) for 20 hr. Expressions of CCR5, CD25, and CD44 on T‐cell (CD3^+^) and their subsets (CD4^+^, CD8^+^, and CD3^+^CD4^−^CD8^−^) cultured with the presence of PGG or placebo. The percentage (%) of the CCR5, CD25, and CD44 positive cells within T‐cell (CD3^+^) compartment and their subsets are indicated. Corresponding values of mean intensity of fluorescence (mean) on individual cell populations are provided (*n* = 7).

* *P* < 0.05.

However, PGG at a dose of 50 μM had no effect on RANTES (CCL5) mRNA expression but effectively decreased the level of chemokine (C–C motif) ligand 2 (CCL2; also referred to as MCP‐1) mRNA in the line of fibroblasts SW872 differentiated to adipocytes (Figure S4a,b), suggesting that the mechanism of this anti‐inflammatory effect is more complex.

### 3.4 PGG inhibits chemokine expression in vivo

Next, we investigated the effect of PGG on chemokine expression in a mouse model of experimental hypertension. Fourteen days of administration of Ang II significantly increased the expression of MCP‐1 and RANTES in both aorta and pVAT (Figure 3b). We also observed increased expression of macrophage inflammatory protein 1 α (MIP‐1α; also known as CCL3) in pVAT following Ang II infusion (Figure 3b). Interestingly, the administration of PGG significantly decreased the expression of both MCP‐1 in aorta and RANTES in pVAT (Figure 3b) while not exerting statistically significant effects on the expression of MIP‐1α and MIP‐1β (also known as CCL4; Figure 3b).

### 3.5 Effect of PGG on T‐cell cytokine production

**Figure 4:**
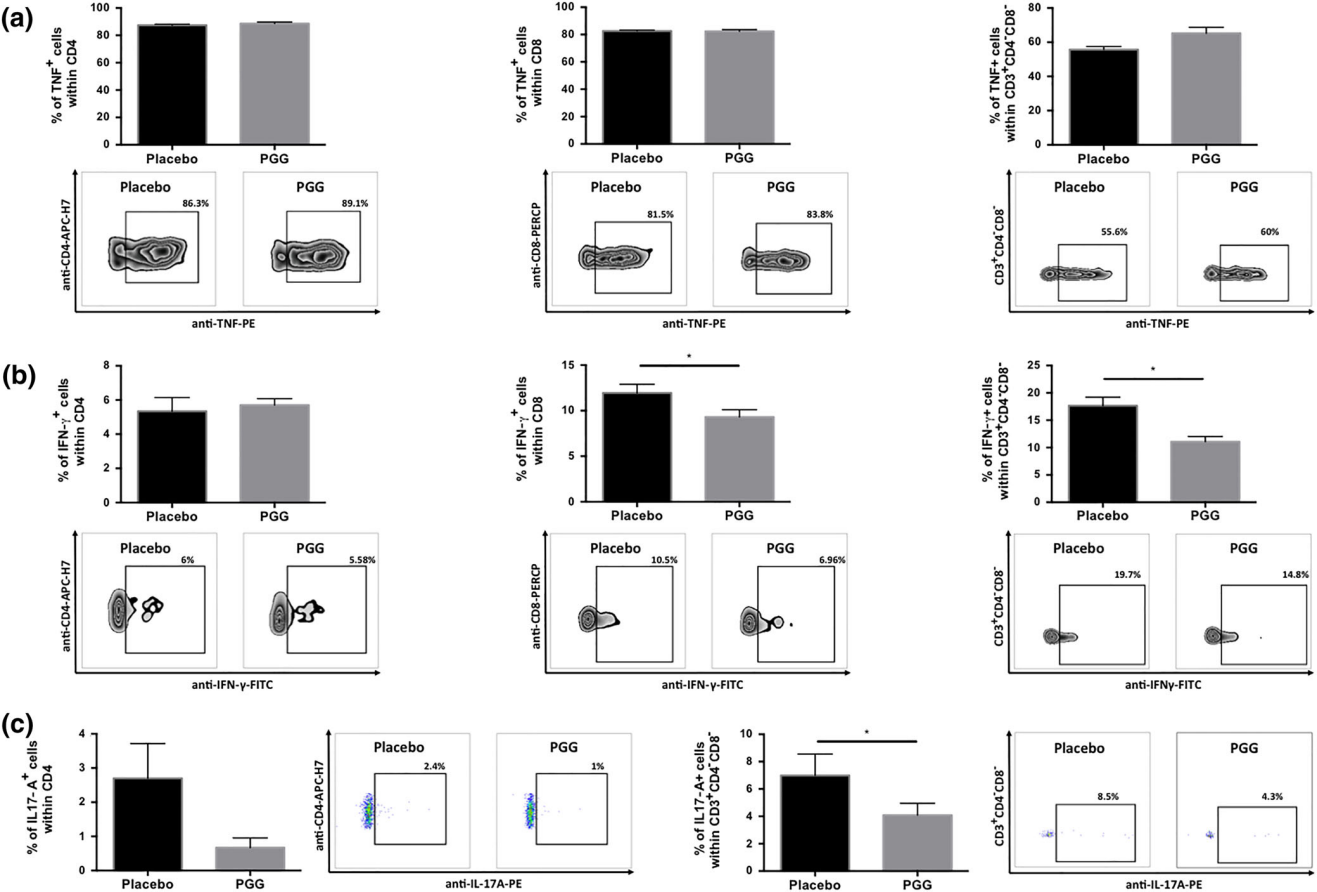
Effect of 1,2,3,4,6‐penta‐*O*‐galloyl‐β‐d‐glucose (PGG) on cytokine production by T‐cells. Freshly isolated splenocytes were stimulated with leukocyte activating cocktail and were cultured in the presence of PGG (final concentration 20 μM) for 4 hr. After this time, intracellular expressions of (a) TNF, (b) IFN‐γ, and (c) IL‐17A within (a–c) CD4^+^, (a, b) CD8^+^, and (a–c) CD3^+^CD4^−^CD8^−^ were assessed by flow cytometry. Average values + SEM and representative flow cytometric examples are shown (*n* = 6). *P < 0.05

Considering the ability of PGG to decrease the expression of some cell activation markers, we next investigated its effect on the production of cytokines by T‐cells. Addition of PGG to T‐cells 30 min prior to stimulation had no effect on the content of CD4^+^TNF^+^, CD8^+^TNF^+^, and CD3^+^CD4^−^CD8^–^TNF^+^ T‐cells (Figure 4a). Using SW872 human fibroblasts differentiated to adipocytes, we have observed no effect of PGG on mRNA encoding TNF expression (Figure S4c). PGG decreased the content of IFN‐γ positive cells, and this effect was particularly seen within CD8^+^ and CD3^+^CD4^−^CD8^−^ T‐cells (Figure 4b). PGG had no effect on the content of CD4^+^IFN‐γ^+^ cells (Figure 4b). PGG administration during stimulation of T‐cells with leukocyte activating cocktail decreased the content of IL‐17A positive CD4^+^ as well as CD3^+^CD4^−^CD8^–^ cells, but this effect was statistically significant only in relation to double negative (CD3^+^CD4^−^CD8^−^) T‐cells (Figure 4c). Additional performed experiments confirmed that PGG in a final concentration of 20 μM did not affect, to a significant degree, the percentage of dead cells in T‐cell culture (data not shown).

### 3.6 Effect of PGG on T‐cell cytokine production in Ang II‐dependent hypertension

**Figure 5:**
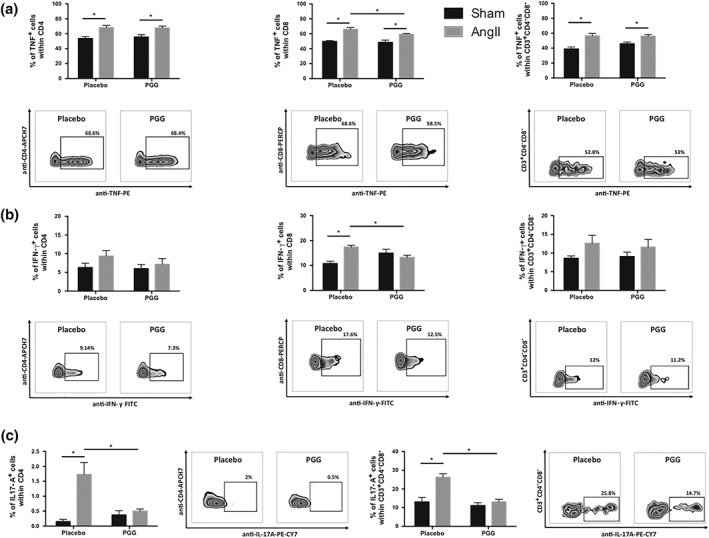
Effect of 1,2,3,4,6‐penta‐*O*‐galloyl‐β‐d‐glucose (PGG) on cytokine production by T‐cells during Ang II‐dependent hypertension. Leukocytes isolated from perivascular adipose tissue of sham‐ and Ang II‐infused mice treated with PGG or placebo were stimulated with leukocyte‐activating cocktail and were cultured for 4 hr. After this time, intracellular expressions of (a) TNF, (b) IFN‐γ, and (c) IL‐17A within (a–c) CD4^+^, (a, b) CD8^+^, and (a–c) CD3^+^CD4^−^CD8^−^ were assessed by flow cytometry. Average values + SEM and representative flow cytometric examples for Ang II‐treated with PGG or placebo are shown (*n* = 5 for sham and *n* = 6–7 for Ang II). *P < 0.05

Next, we examined the effect of PGG on cytokine production by T‐cells isolated from pVAT from sham‐ and Ang II‐treated mice. Chronic Ang II administration increased the number of CD4^+^TNF^+^, CD8^+^TNF^+^, and CD3^+^CD4^−^CD8^−^TNF^+^ T‐cells in either PGG‐treated or placebo group (Figure 5a). Interestingly, an i.p. administration of PGG (every 2 days) starting 1 week before minipump implantation and throughout the Ang II infusion significantly reduced the number of CD8^+^TNF^+^ cells but had no effect on CD4^+^TNF^+^ and CD3^+^CD4^−^CD8^−^TNF^+^ T‐cells (Figure 5a). Ang II administration significantly increased the number of CD8^+^IFN**‐**γ^+^ T‐cells, and PGG administration decreased the content of these cells (Figure 5b).

Ang II infusion increased the content of CD4^+^IL‐17A^+^ and CD3^+^CD4^−^CD8^−^IL‐17A^+^ T‐cells. This effect was not observed in the group treated with PGG. Interestingly, the administration of PGG significantly reduced the content of IL‐17A positive CD4^+^ as well as CD3^+^CD4^−^CD8^−^ T‐cells (Figure 5c).

### 3.7 Effect of PGG on vascular dysfunction and BP elevation in response to Ang II

**Figure 6:**
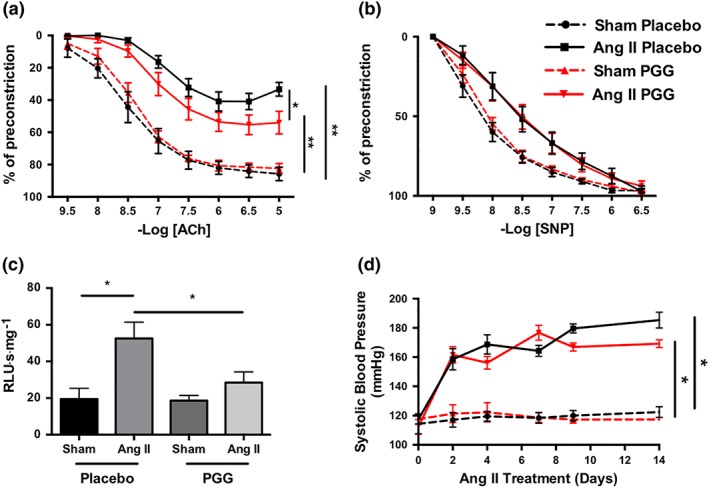
Effect of 1,2,3,4,6‐penta‐*O*‐galloyl‐β‐d‐glucose (PGG) on Ang II‐dependent hypertension and regulation of vascular dysfunction. (a) Effect of PGG on endothelium‐dependent vasodilatation to ACh in aortas of mice infused with Ang II (*n* = 7 for sham and *n* = 10 for Ang II). (b) Endothelium independent vasodilatation shown as relaxations to sodium nitroprusside after administration of PGG (*n* = 7 for sham and *n* = 10 for Ang II). Statistical analysis was performed by repeated‐measures ANOVA. (c) Aortic superoxide production measured by lucigenin‐enhanced chemiluminescence (5 μM) in mice infused for 14 days with buffer (sham) or Ang II and treated with PGG or placebo (*n* = 12 each group). (d) Mean daily values of non‐invasive BP measurements by tail‐cuff plethysmography during Ang II infusion in mice treated with PGG or placebo (*n* = 6 each group). *P < 0.05

We next investigated the effect of PGG on vascular function, ROS production, and BP regulation in the context of alterations to inflammatory processes previously linked to hypertensive pathology. Importantly, PGG partially prevented development of endothelial dysfunction evoked by Ang II‐induced hypertension (Figure 6a), while endothelium‐independent responses to SNP were not altered (Figure 6b). In line with this, PGG prevented vascular oxidative stress as measured by lucigenin‐enhanced chemiluminescence in aortic rings (Figure 6c). Interestingly, these changes were independent of BP regulation as Ang II increased BP to the same extent in both placebo‐treated and PGG‐treated mice (Figure 6d).

## 4 DISCUSSION

As we are learning about the potential benefits of limiting vascular inflammation (Kusters et al., 2018; Ridker et al., 2017), there is an urgent need to identify novel ways to target vascular inflammation and investigate their effects on cardiovascular parameters. PGG represents a promising candidate in the prevention and therapy of many inflammatory diseases, diabetes, and cancer (Zhang et al., 2009). While numerous studies have focused on its antitumour activity in a number of pathologies, primarily through the induction of apoptosis of cancer cells, several key papers have suggested significant anti‐inflammatory properties of PGG (Feldman et al., 2001; Kang et al., 2005; Kiss, Filipek, Zyzynska‐Granica, & Naruszewicz, 2013; Wu & Gu, 2009). In the present study, we focused on specific effects of PGG on vascular inflammation, showing that it affects, in particular, cellular components of the inflammatory infiltrate and may also inhibit T‐cell activation and recruitment to the vessel wall. This is important in the prevention of vascular damage as a consequence of hypertension, which we have used as a model system of vascular inflammation. In preliminary experiments, we observed that PGG decreased the number of T‐cells in pVAT while having only a moderate effect on macrophages. For this reason, we have focused on characterization of T‐cell subsets in more details. In addition, this direction of study follows our previous investigations showing the effect on T‐cells on vascular function in hypertension (Mikolajczyk et al., 2016), while macrophages were demonstrated to be more important in models of atherosclerosis (Skiba et al., 2017).

Our studies are in line with previous reports of systemic anti‐inflammatory activity of PGG, which suppresses a number of pro‐inflammatory cytokines (such as TNF‐α) from human peripheral blood mononuclear cells exposed to LPS (Feldman et al., 2001). Through the suppression of TNF‐α, PGG effectively protects from otherwise lethal doses of LPS challenge (Genfa et al., 2005). PGG significantly reduced LPS‐induced NO production in macrophages, decreased gene expression and production of iNOS in a dose‐dependent manner (Kim et al., 2009; Lee, Lee, & Mar, 2003), and inhibited COX‐2 activity in LPS‐activated macrophages (Lee et al., 2003) as well as the activation of NF‐κB (Kim et al., 2009). In addition, PGG, inhibited gene expression and secretion of IL‐8, a major mediator of acute neutrophil‐mediated inflammation, and CCL‐2, a major mediator of chronic macrophage‐mediated inflammation in human monocytic U937 cells stimulated with phorbol myristate acetate (Oh et al., 2003).

However, no studies to date have addressed the effect of this compound on vascular inflammation and cytokine production, particularly essential for the regulation of endothelial function. We have recently shown that IFN‐γ produced by CD8 cells infiltrating pVAT appears to be particularly important in the regulation of vascular function in hypertension (Kossmann et al., 2013; Mikolajczyk et al., 2016). This is essential because endothelial dysfunction precedes development of atherosclerosis and leads to the clinical complications of hypertension (Wilk et al., 2013).

In the present study, we show for the first time that 20 μM of PGG suppressed PMA‐ and calcium ionophore‐induced release of IFN‐γ from both CD8^+^ and CD3^+^CD4^−^CD8^−^ cells.

Interestingly, while PGG administration affected a variety of immune cells and pro‐inflammatory mechanisms, there was a marked decrease in the production of IFN‐γ in CD8^+^ T‐cells upon Ang II infusion in comparison to the same group treated with placebo.

While PGG supressed other markers of T‐cell activation, it did not induce cell death in the examined T‐cells.

Our findings are in line with recent studies by Kim et al. (2015) who observed that p.o. administration of PGG suppressed the production of T helper 2 (IL‐4 and IL‐13) and T helper 1 (IFN‐γ) as well as pro‐inflammatory cytokines such TNF‐α and IL‐6. However, this was not observed in the case of anti‐inflammatory IL‐10 in ovalbumin‐restimulated splenocytes from ovalbumin‐sensitized mice.

Interestingly, PGG selectively induced IL‐10 production in serum related to regulatory T‐cells (Kim et al., 2015). Effects of PGG on macrophages are better characterized. PGG has been shown to suppress IL‐1β, TNF‐α, and IL‐6 in LPS‐stimulated peritoneal macrophages while increasing expression of the anti‐inflammatory cytokine IL‐10. This effect was mediated by suppression of NF‐κB and MAPK signalling pathways in a MyD88 adaptor protein‐dependent manner (Jang, Hyam, Jeong, Han, & Kim, 2013).

Although we did not observe a decreased in vitro production of TNF‐α after PGG administration in neither T‐cells nor SW872‐derived adipocytes, such an effect was evident in response to in vivo administration of PGG. PGG significantly reduced the number of CD8^+^TNF^+^ cells but had no effect on the content of CD4^+^TNF^+^ and CD3^+^CD4^−^CD8^−^TNF^+^ T‐cells.

Previous studies have demonstrated an essential role of IL‐17 in hypertensive vascular dysfunction (Madhur et al., 2010); (Saleh, Norlander, & Madhur, 2016). Therefore, we investigated the effects of PGG on the expression of this cytokine upon activation. While we observed the suppression of IL‐17 production across all T‐cell subsets in vitro, it reached statistical significance only in CD3^+^CD4^−^CD8^−^ cells, which is in line with the fact that these cells are the predominant source of IL‐17 in hypertension (Caillon et al., 2017; Saleh et al., 2016). In vivo study using an experimental model of Ang II‐dependent hypertension confirmed that PGG also effectively inhibited the production of IL‐17A by CD4^+^ and CD3^+^CD4^−^CD8^−^ T‐cell subsets. Interestingly, while we observed a number of vasoprotective effects in relation to vascular inflammation, endothelial dysfunction, and vascular oxidative stress, these effects were independent of BP regulation, as i.p. injection of PGG at a dose of 10 mg·kg^−1^ every 2 days did not attenuate Ang II‐induced hypertension. There was, however, a modest trend towards a mild BP reduction in these mice. It is also important to note that we have only performed tail‐cuff BP measurement, and the use of telemetry could result in a more sensitive effect. Lack of antihypertensive effect may also be linked to the model we used. Interestingly, PGG revealed a strong dose‐dependent hypotensive effect, reducing the BP significantly in spontaneously hypertensive rats with infusion of angiotensin I (Liu et al., 2003). However, in that study, the authors used a complex model of spontaneous hypertension (through the use of spontaneously hypertensive rats), which is more sophisticated than the model of Ang II‐induced hypertension (Lin, Lee, Chan, & Tse, 2016; Liu et al., 2003). It should be emphasized that in that study, the authors also investigated the acute effect of PGG preceded by an infusion of Ang I. In contrast, we focused on the chronic effects of PGG, which did not reduce BP response to Ang II in our study.

ROS plays an important role in the regulation of endothelial function (Radziwon‐Balicka et al., 2017; Shafique et al., 2017; Tsai et al., 2017). It is well known that PGG acts as a ROS scavenger (Viswanatha, Shylaja, & Mohan, 2013). Moreover, recently published studies have revealed that PGG induced SOD activity in Caenorhabditis elegans and reduced intracellular ROS accumulation in a dose‐dependent manner (Ahn et al., 2013). Early studies on PGG have demonstrated that it can protect cells from oxidative stress via heme oxygenase‐1 induction, indicating that the PGG can also act as an indirect antioxidant (Choi et al., 2002). The effects of PGG on vascular function are important. Although they may be partially linked to the reduction of vascular inflammation, PGG may also have direct effects on the vasculature. Indeed, we observed a significant reduction of vascular oxidative stress by PGG, in line with the fact that tannins have antioxidant properties (Riedl & Hagerman, 2001; Viswanatha et al., 2013) in a number of cellular models (Abdelwahed et al., 2007; Okubo et al., 2000).

It is likely that the effects on endothelial function are mediated in part by the direct antioxidant properties of PGG, leading to an increase of NO bioavailability and in part indirectly, by reducing perivascular inflammation. The latter mechanism is supported by our previous study showing that perivascular T‐cell infiltration may stimulate both oxidative stress and endothelial function (Mikolajczyk et al., 2016). In that study, we demonstrated that IFN**‐γ** was pivotal in the induction of endothelial dysfunction (Mikolajczyk et al., 2016). Interestingly, PGG affected T‐cell IFN‐**γ** production to the largest extent in the present in vivo study. This possible pleiotropic effect may indicate that compounds such as PGG may target multiple mechanisms of vascular dysfunction and disease, which could make them particularly valuable in multifactorial diseases such as hypertension. We observed that PGG normalized ROS production but only modestly improved vascular function. However, in our study, we used lucigenin‐enhanced chemiluminescence, which allows detection of superoxide anion production. It is known that other radicals (hydroxyl radical, lipid peroxyl radical, and alkoxyl radical) and other molecules including peroxynitrite, hypochlorous acid, and hydrogen peroxide have strong oxidant properties and can affect vascular function (Guzik et al., 2002). Furthermore, Ang II and H_2_O_2_ cause the phosphorylation of eNOS on Tyr657, attenuating NO production (Carnicer et al., 2017; Douglas et al., 2018; Loot, Schreiber, Fisslthaler, & Fleming, 2009). Finally, Ang II could cause an H4B deficiency leading to eNOS uncoupling, resulting in lower NO production. All factors mentioned above are likely to work in concert, leading to the impairment of endothelium‐dependent vasodilatation in hypertensive animals.

We observed that in response to SNP, there was a rightward shift in Ang II‐treated aortas. This was seen in both PGG and placebo groups. This phenomenon is common in vascular studies of aortas from Ang II‐infused animals and was observed previously by our group as well as others (Itani et al., 2016; Madhur et al., 2010; Mikolajczyk et al., 2016; Siedlinski et al., 2017) and may be linked to the impaired sensitivity of vascular smooth muscle cells and soluble guanylyl cyclase to NO upon chronic Ang II infusion (Sorop et al., 2017). Reduction of vascular oxidative stress and endothelial dysfunction may serve as important mechanisms in the reduction of vascular inflammation as both chemokines and adhesion molecules are redox sensitive. Indeed, in a recent study, PGG was shown to inhibit a number of pro‐inflammatory mediators such as LTB4, IL‐8, and myeloperoxidase in human neutrophils through the suppression of ROS, particularly O_2_^−^ (Kiss et al., 2010). PGG is also known to inhibit β2‐integrin (CD11b) and L‐selectin (CD62L) expression on leukocytes (Kiss et al., 2013). Our findings support and extend these observations, showing reduced expression of molecules critical for the development of perivascular inflammation including CCR5 and CD44. This resulted in significantly decreased total leukocyte and T‐cell infiltration in pVAT.

Experiments performed by our group on a human line of SW872 fibroblasts differentiated to adipocytes revealed that PGG at a dose of 50 μM effectively decreased the level of CCL‐2 mRNA while having no effect on RANTES expression. However, the administration of PGG decreased RANTES mRNA level in pVAT during Ang II‐dependent hypertension, which could be associated with decreased T‐cells and infiltration by their subsets (especially CD4^+^CCR5^+^ and CD8^+^CCR5^+^) into the pVAT. We also observed decreased expression of MCP‐1 after PGG treatment in Ang II‐infused aorta, but this effect was not seen in pVAT. This finding can be related to the moderate effect of PGG on macrophage contents in pVAT. While we have only focused on a limited number of mechanisms known to be particularly linked to hypertensive vascular dysfunction, Kang et al. (2005) have previously shown that PGG suppresses the expression of adhesion molecules such as endothelial intracellular cell adhesion molecule‐1 and vascular cell adhesion molecule‐1 induced by TNF‐α in concert with reduced CCL‐2 expression.

In summary, our results suggest that PGG is an important candidate for targeting vascular inflammation through both vascular effects (protection from vascular oxidative stress and endothelial dysfunction) and direct effects on T‐cell activation and their perivascular recruitment. Thus, PGG, may provide opportunities for the effective treatment of hypertension.

## CONFLICT OF INTEREST

The authors declare no conflicts of interest.

## AUTHOR CONTRIBUTIONS

T.P.M. designed and conducted the majority of in vivo and in vitro experiments, analysed the data, prepared figures, and wrote the manuscript; R.N. performed vascular experiments and participated in data analysis; D.S. performed in vitro experiments on adipocytes; J.K. performed tail‐cuff BP measurements and sample preparation for flow cytometry; M.M. performed tail‐cuff BP and ROS measurements; A.S.J‐J. took part in RNA isolation and gene expression measurements; P.K. took part in partial ROS measurements; Z.K. took part in partial ROS measurements; A.S‐L. performed tail‐cuff BP measurements; K.L. critically revised the manuscript; P.M. made intellectual contributions to the manuscript; D.G. contributed to in vivo experiment and made intellectual contributions to the manuscript; A.K. performed PGG isolation and characterization and made intellectual contributions to the manuscript; M.N. revised critically for intellectual contribution to the manuscript; T.J.G. conceived, designed, and supervised experiments, obtained funding, and wrote the manuscript.

## DECLARATION OF TRANSPARENCY AND SCIENTIFIC RIGOUR

This Declaration acknowledges that this paper adheres to the principles for transparent reporting and scientific rigour of preclinical research as stated in the *BJP* guidelines for Design & Analysis, Immunoblotting and Immunochemistry, and Animal Experimentation, and as recommended by funding agencies, publishers and other organisations engaged with supporting research.

## Supporting information


**Figure S1**
HPLC‐DAD chromatograms of crude seed extract (**A**) and isolated PGG (**B**) recorded at 280 nm; UV and MS data of isolated PGG (**C**).

**Figure S2**
^**1**^ **H (A),** ^**13**^ **C (B)**, spectra of PGG.

**Figure S3**
**Effect of PGG on leukocyte infiltration in visceral adipose tissue during Ang II–dependent hypertension.** Hypertension was induced by chronic 14‐day Ang II infusion by osmotic minipump (490 ng·min^−1^ kg^−1^) and leukocytes were obtained from visceral adipose tissue (VAT) by enzymatic digestion. **A)** Effect of Ang II infusion and PGG administration on absolute number of CD45+ leukocytes in VAT expressed per mg of tissue (*n* = 7). **B)** and **C)** Effect of Ang II infusion and PGG administration on CD3+ T cells (n = 7) and F4/80 + CD11b + macrophages (*n* = 5) content, respectively.

**Figure S4**
**Effect of PGG on the expression of selected genes.** Human line of SW872 fibroblasts differentiated to adipocytes were stimulated with PGG at a dose of 50 μM for 24 hrs. After this time, RNA was isolated and the expression of A) RANTES, B) MCP‐1, C) TNF‐α was assessed (*n* = 4). **‐ *p* < 0.01
