# Supplementary figures and images for: 1,2,3,4,6‐Penta‐O‐galloyl‐β‐d‐glucose modulates perivascular inflammation and prevents vascular dysfunction in angiotensin II‐induced hypertension
Source: Br J Pharmacol. 2019 Mar 14;176(12):1951–65. doi: 10.1111/bph.14583 (PMC6534792; doi:10.1111/bph.14583)

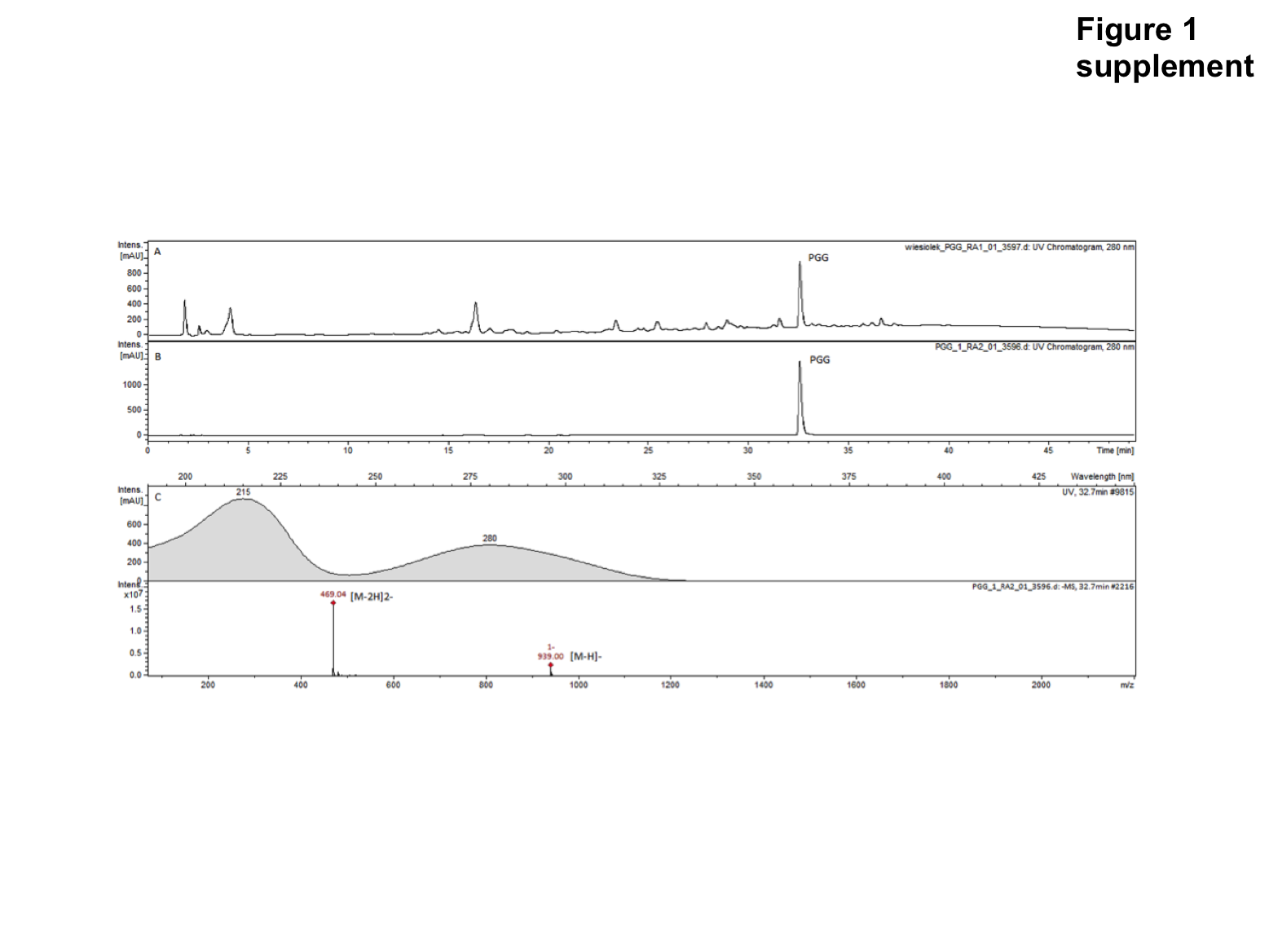

Supplement: Supplementary file 1 — Figure S1 HPLC‐DAD chromatograms of crude seed extract (A) and isolated PGG (B) recorded at 280 nm; UV and MS data of isolated PGG (C). [file BPH-176-1951-s001.tiff]

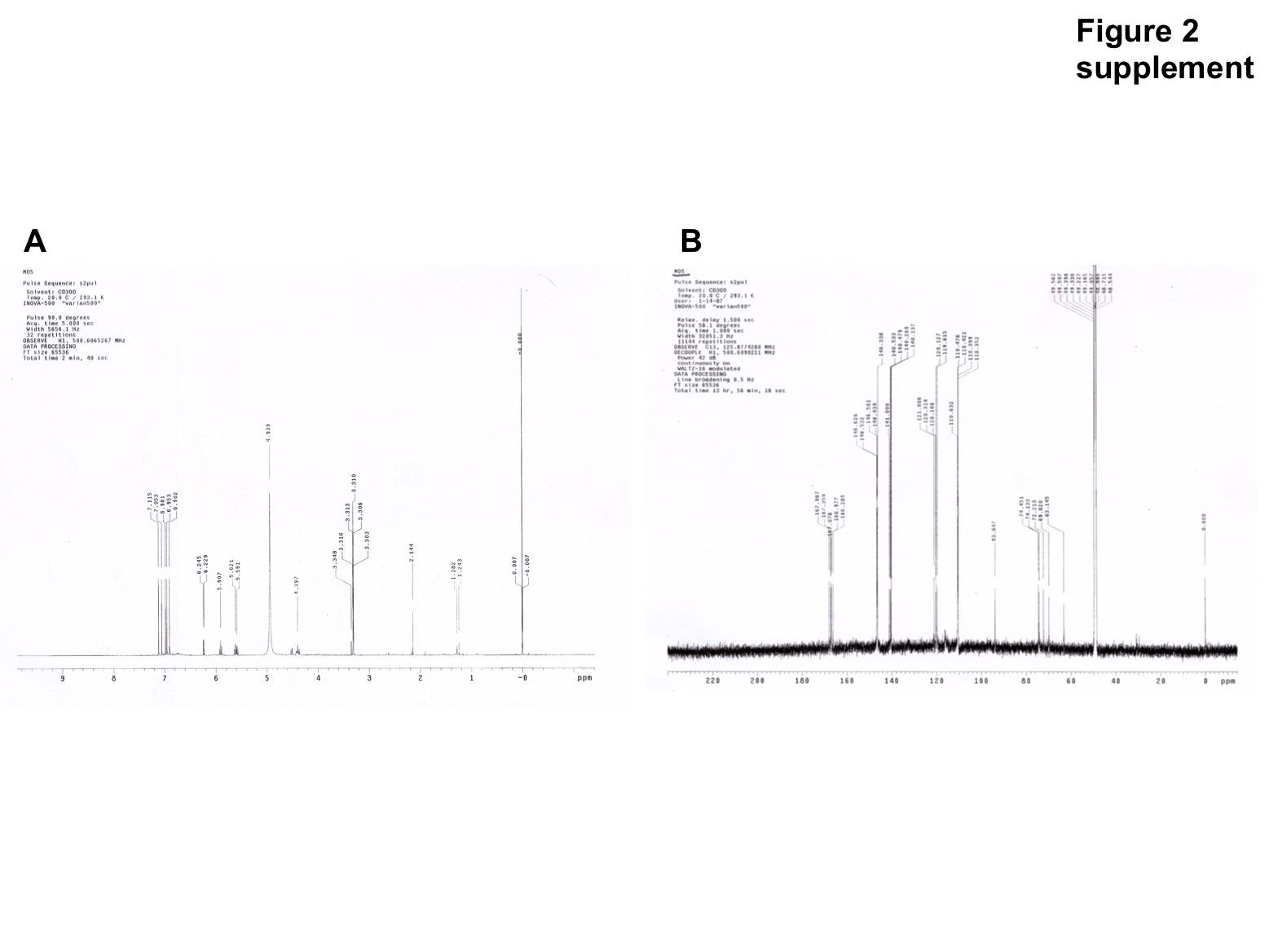

Supplement: Supplementary file 2 — Figure S2 1 H (A), 13 C (B), spectra of PGG. [file BPH-176-1951-s002.tiff]

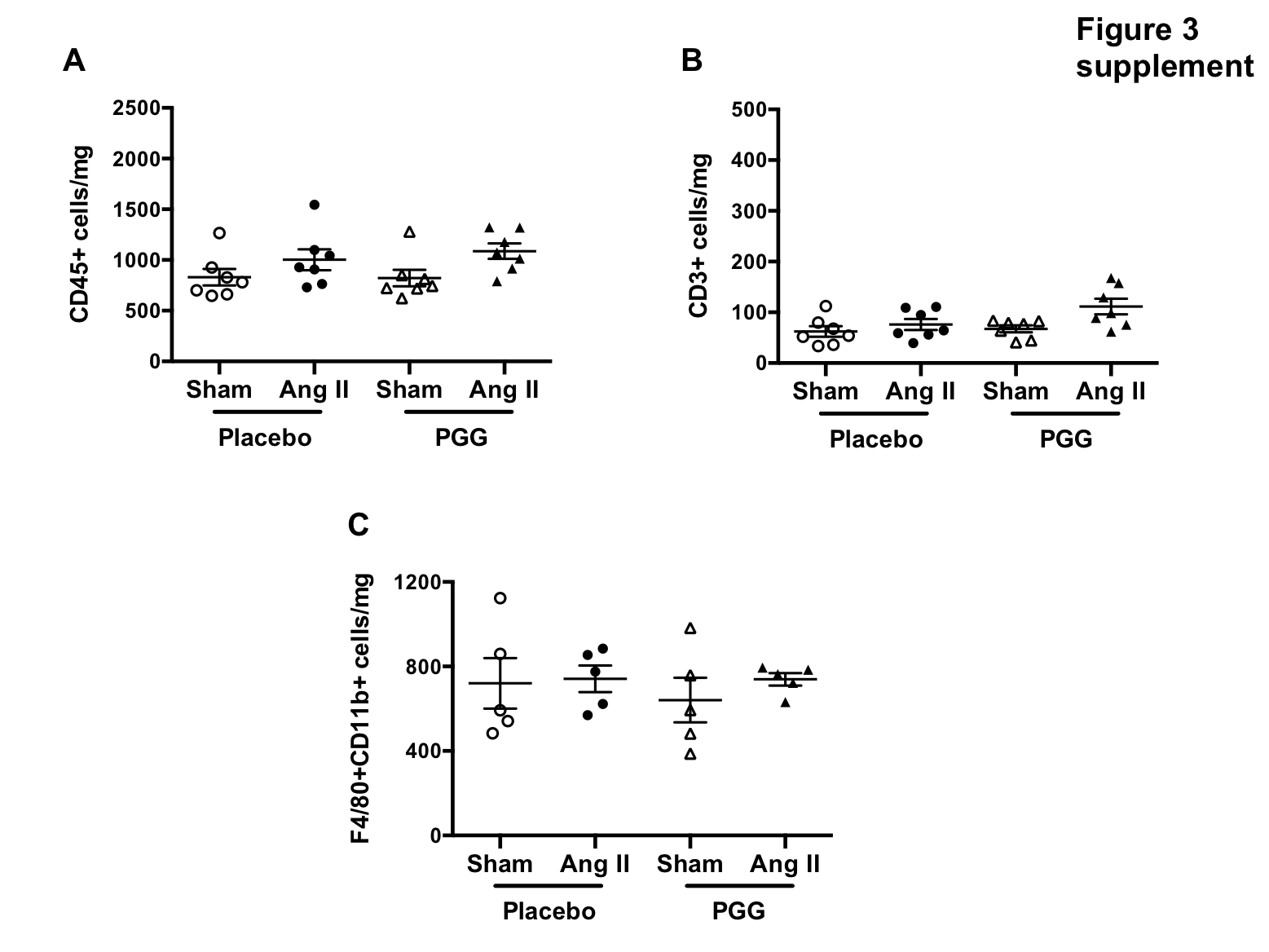

Supplement: Supplementary file 3 — Figure S3 Effect of PGG on leukocyte infiltration in visceral adipose tissue during Ang II–dependent hypertension. Hypertension was induced by chronic 14‐day Ang II infusion by osmotic minipump (490 ng·min−1 kg−1) and leukocytes were obtained from visceral adipose tissue (VAT) by enzymatic digestion. A) Effect of Ang II infusion and PGG administration on absolute number of CD45+ leukocytes in VAT expressed per mg of tissue (n = 7). B) and C) Effect of Ang II infusion and PGG administration on CD3+ T cells (n = 7) and F4/80 + CD11b + macrophages (n = 5) content, respectively. [file BPH-176-1951-s003.tiff]

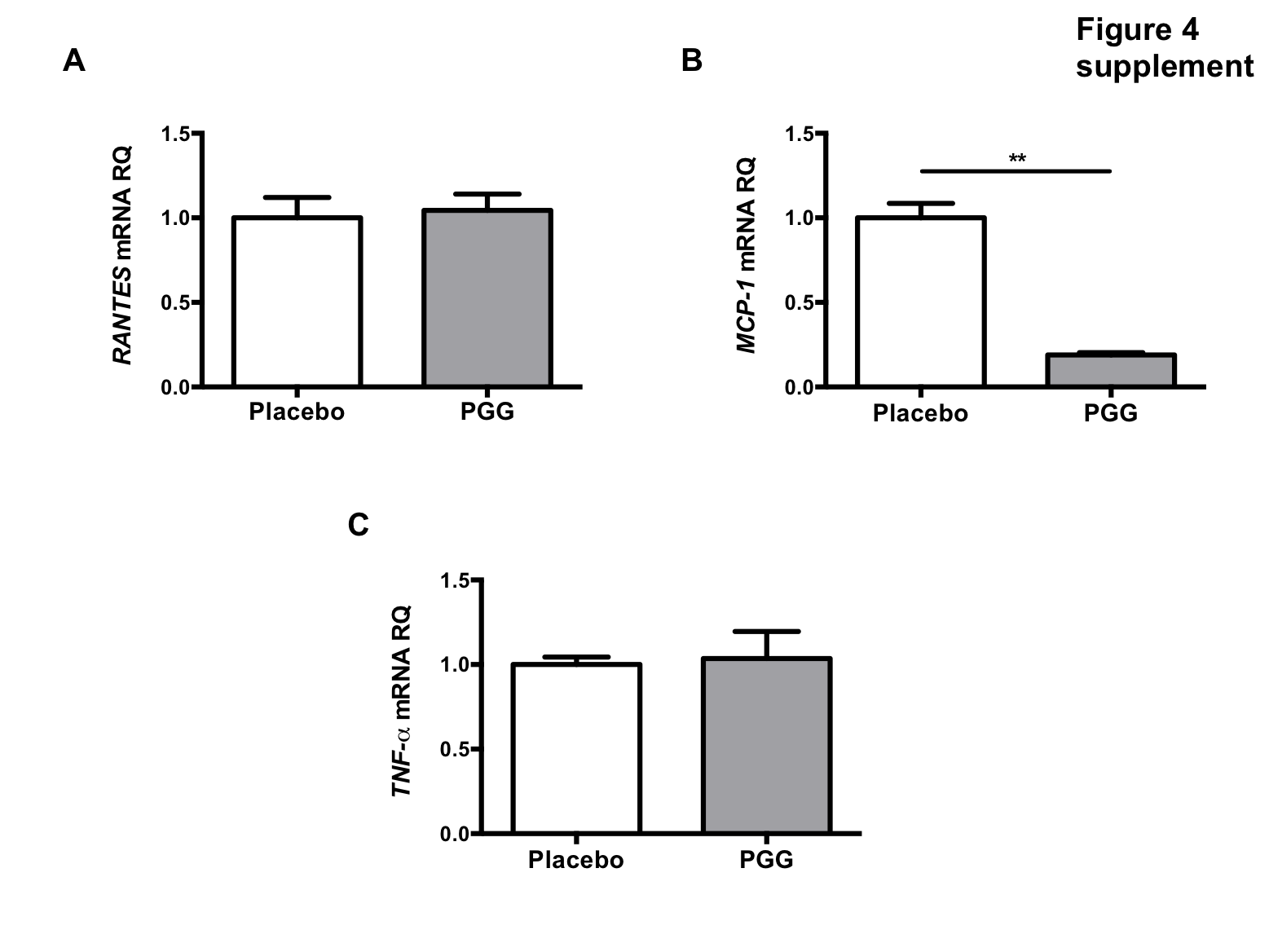

Supplement: Supplementary file 4 — Figure S4 Effect of PGG on the expression of selected genes. Human line of SW872 fibroblasts differentiated to adipocytes were stimulated with PGG at a dose of 50 μM for 24 hrs. After this time, RNA was isolated and the expression of A) RANTES, B) MCP‐1, C) TNF‐α was assessed (n = 4). **‐ p < 0.01 [file BPH-176-1951-s004.tiff]
